# Supramolecular Ion-Channel Engineering of Spin–Charge Coexistence in a [Ni(dmit)_2_] Conductor Hosting Mixed-Valence Mn Cations

**DOI:** 10.1021/acs.inorgchem.6c00118

**Published:** 2026-02-05

**Authors:** Daisuke Ishikawa, Jun Manabe, Masato Haneda, Kiyonori Takahashi, Takayoshi Nakamura, Sadafumi Nishihara

**Affiliations:** † Department of Chemistry, Graduate School of Advanced Science and Engineering, 12803 Hiroshima University, 1-3-1, Kagamiyama, Higashi-hiroshima, Hiroshima 739-8526, Japan; ‡ Department of Chemistry, 13205 Kumamoto University, 2-39-1 Kurokami, Chuo-ku, Kumamoto 860-8555, Japan; § Research Institute for Electronic Science, Hokkaido University, N20W10, Kita-ku, Sapporo 001-0020, Japan; ∥ Chirality Research Center (CResCent), Hiroshima University, 1-3-1, Kagamiyama, Higashi-hiroshima, Hiroshima 739-8526, Japan; ⊥ Precursory Research for Embryonic Science and Technology (PRESTO), Japan Science and Technology Agency, 4-1-8, Honcho, Kawaguchi, Saitama 332-0012, Japan

## Abstract

The interplay between electrical conduction and magnetism offers a powerful means to elucidate emergent mechanisms and control properties; however, realizing this in Ni­(dmit)_2_ crystals has been challenging due to undesirable reactions among their components. Mn_1.83_([18]­crown-6)_3_[Ni­(dmit)_2_]_11_(H_2_O)_7.33_(CH_3_CN)_2_ (**1**) is prepared in the present study, integrating one-dimensional [18]­crown-6 ion channels hosting mixed-valence Mn^2+^/Mn^3+^ with conducting [Ni­(dmit)_2_] layers. Subsequently, a structure-driven mechanism of conductivity is clarified. In the crystal, [Ni­(dmit)_2_] forms one-dimensional dimer–dimer–trimer–dimer–dimer stacks; weak interchain contacts generate two-dimensional sheets alternating with supramolecular channel layers. Mn ions occupy two partially populated sites and adopt seven-coordinate environments with two axial aqua ligands and five equatorial crown-ether oxygen. Magnetometry indicates Mn moments are effectively decoupled from the conducting [Ni­(dmit)_2_] sublattice: the Mn sublattice follows Curie–Weiss behavior with an exceptionally small Weiss temperature, while the [Ni­(dmit)_2_] stacks form *S* = 1/2 one-dimensional Heisenberg antiferromagnetic chains. Compound **1** exhibits high conductivity at 300 K and one-dimensional variable-range hopping, attributable to thermal fluctuations of the supramolecular channels that modulate intracolumn transfer integrals and promote carrier localization. To our knowledge, **1** is the first system combining transition-metal-ion [18]­crown-6 channels with conducting [Ni­(dmit)_2_] layers, establishing a supramolecular route to tune spin–charge coexistence via host design.

## Graphical abstract

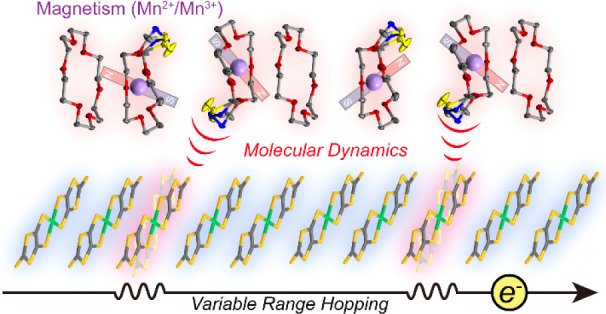

## Introduction

The cooperative interplay between electrical conduction and magnetism gives rise to a broad range of physical phenomena, many of which are technologically significant. The key control parameter is the coupling strength between charge carriers in metals and semiconductors (hereafter, “carriers”) and localized spins in solids.^1^ When spins are strongly coupled to the carriers, the exchange field substantially polarizes the electronic bands, leading to functionalities such as colossal magnetoresistance,^2^ spin-polarized transport,^3^ and the anomalous and topological Hall effects.^4,5^ In contrast, when spin–carrier coupling is weak, long-range Ruderman–Kittel–Kasuya–Yosida (RKKY) interactions are expected, and phenomena associated with broken inversion symmetry, such as magnetochiral anisotropy, can emerge.^6,7^ Moreover, even when the two subsystems coexist while decoupled, cooperative conduction and magnetism can still appear. For example, in noncontact magnetic gating, which is used as a wiring-free resistive switch and as a differential gate, the application of a weak in-plane magnetic field modulates the resistance^8^ because magnetization produced by spin alignment deflects the current via the Lorentz force.

The cooperative interplay between electrical conduction and magnetism has also attracted attention in molecular crystals, a platform well-suited for elucidating structure–property correlations. The deliberate introduction of magnetic ions into superconducting crystals has enabled systematic studies on the correlations between localized spins and itinerant electrons, and on concomitant property control. In particular, the isostructural, λ-type salts of BETS (bis­(ethylenedithio)­tetraselenafulvalene), λ-(BETS)_2_MCl_4_ (M = Ga, Fe), have been examined in detail by comparing the nonmagnetic GaCl_4_^–^ and magnetic FeCl_4_^–^ analogues. λ-(BETS)_2_GaCl_4_ exhibits a superconducting transition at 5.5 K under ambient pressure, whereas λ-(BETS)_2_FeCl_4_ exhibits an antiferromagnetic insulating state at low temperature, driven by strong π-*d* exchange coupling between the Fe^3+^ localized moments and the π-electrons in the BETS layers. Moreover, a magnetic-field-induced superconducting phase attributed to the Jaccarino–Peter effect has been observed, in which the exchange field arising from polarized Fe^3+^ moments compensates the external Zeeman field and mitigates paramagnetic pair breaking.^9,10^

The [Ni­(dmit)_2_]^−^ complex^11^ (dmit^2–^ = 1,3-dithiole-2-thione-4,5-dithiolate) is an open-shell molecule carrying an *S* = 1/2 spin. Upon partial oxidation, this complex forms highly conducting molecular crystals, and superconducting transitions are observed not only in charge-transfer complexes, such as α-(EDT-TTF)­[Ni­(dmit)_2_] (EDT-TTF = ethylenedithiotetrathiafulvalene) (*T*_c_ = 1.3 K) and TTF­[Ni­(dmit)_2_]_2_ (TTF = tetrathiafulvalene) (*T*_c_ = 1.62 K at 0.7 GPa), but also in anion-radical salts such as (CH_3_)_4_N­[Ni­(dmit)_2_]_2_ (*T*_c_ = 5.0 K at 0.7 GPa).^12–14^ [Ni­(dmit)_2_]-based anionic molecular conductors are counterparts to the cationic systems represented by BEDT-TTF and BETS. They provide an equally important platform for investigating diverse electronic functionalities and structure–property correlations, particularly from the perspective of the cooperative interplay between conduction and magnetism, because transition-metal cations with magnetic moments are employed directly as counter cations. Furthermore, metallic conduction accompanied by an antiferromagnetic transition has been reported in the DCNQI-based mixed-valence conductor (DMDCNQI)_2_Cu, providing a precedent for the coexistence of charge transport and magnetic order in π-acceptor-based molecular conductors.^15,16^

However, incorporating magnetic transition-metal ions into [Ni­(dmit)_2_]-based salts is highly challenging. Electrocrystallization and related procedures used to obtain partially oxidized salts often induce undesired reactions between the transition-metal ions and the dmit^2–^ ligand, which produce complex reaction mixtures. Although crystals can be grown by employing stable molecular species, such as metal complexes as counter cations, the intrinsic properties of metal complexes tend to dominate, making it difficult to achieve the desired cooperative interplay between magnetism and electrical conduction. It is therefore desirable to incorporate transition-metal ions into the crystal in a weakly coordinating environment. To suppress coordination or ligand-exchange reactions with the dmit^2–^ ligand, crown ethers or related hosts can be introduced into the reaction medium to encapsulate the transition-metal ions and sterically protect them from side reactions. This strategy allows the incorporation of transition-metal ions into Ni­(dmit)_2_ crystals and, simultaneously, realizes the coexistence of functionalities derived from the structural flexibility of the supramolecular cations.^17,18^

In a previous study, the authors constructed supramolecular cation architectures within conducting [Ni­(dmit)_2_]-based crystals by combining [18]­crown-6 with alkali metal cations.^19^ In the resulting crystals of M^+^_*x*_([18]­crown-6)­[Ni­(dmit)_2_]_2_ (*x* < 1), the [18]­crown-6 molecules form one-dimensional channel structures. The dynamic state and coordination environment of cations in these channels depend strongly on the ionic size. This size dependence significantly affects electrical transport. For example, the Li^+^ salt shows metallic conductivity and Li^+^ motion along the channels at high temperatures (200–300 K). When ion transport stops at low temperatures, the electronic system becomes semiconducting. In the Cs^+^ salt, the Cs^+^ ions are sandwiched by [18]­crown-6 molecules and adopt an incommensurate periodic arrangement. As a result, the compound exhibits fluctuations characteristic of a charge density wave.

This study employs crown ethers to synthesize new crystals in which the magnetic Mn ions and Ni­(dmit)_2_ coexist, with the aim of elucidating the correlations between the structure and physical properties. In the resulting crystals, Mn_1.83_([18]­crown-6)_3_[Ni­(dmit)_2_]_11_(H_2_O)_7.33_(CH_3_CN)_2_ (**1**), the [18]­crown-6 molecules assemble into channel structures, within which Mn^2+^ and Mn^3+^ ions coexist. [Ni­(dmit)_2_] forms a one-dimensional column structure consisting of (dimer–dimer–trimer–dimer–dimer) stacking sequences, a motif that has not yet been reported. The magnetic behavior is explained by a one-dimensional Heisenberg antiferromagnetic chain for the [Ni­(dmit)_2_] columns and Curie–Weiss spins for the Mn ions. The crystal exhibits high room-temperature conductivity (4.7 S cm^–1^) with a temperature dependence consistent with one-dimensional variable range hopping (VRH). This behavior is attributed to thermal fluctuations of the supramolecular channels, which modulate the [Ni­(dmit)_2_] columns to localize the electronic wave functions.

## Results and Discussion

Figure 1 shows the crystal structure of **1** at 100 K. Compound **1** crystallizes in the triclinic system with space group *P*1̅. The asymmetric unit contains one crystallographically independent Mn ion, one [18]­crown-6 molecule (**CE1**), one-half of a [18]­crown-6 molecule (**CE2**) located on an inversion center, five independent [Ni­(dmit)_2_] anions (labeled **A**–**E**), one-half of an [Ni­(dmit)_2_] anion (**F**) located on an inversion center, five water molecules, and one acetonitrile molecule (Figure S1). In crystal **1**, the [Ni­(dmit)_2_] anions form one-dimensional chains, which extend into two-dimensional layers alternately stacked with supramolecular layers along the *c* axis (Figure 1a). Within the supramolecular layers, ion channels composed of Mn_1.83_([18]­crown-6)_3_(H_2_O)_7.33_ are formed. These channels extend in a direction that makes an angle of approximately 80°with the one-dimensional [Ni­(dmit)_2_] columns. Acetonitrile molecules are located between neighboring channels, which effectively isolates the channels (Figure 1b).

**Figure 1:**
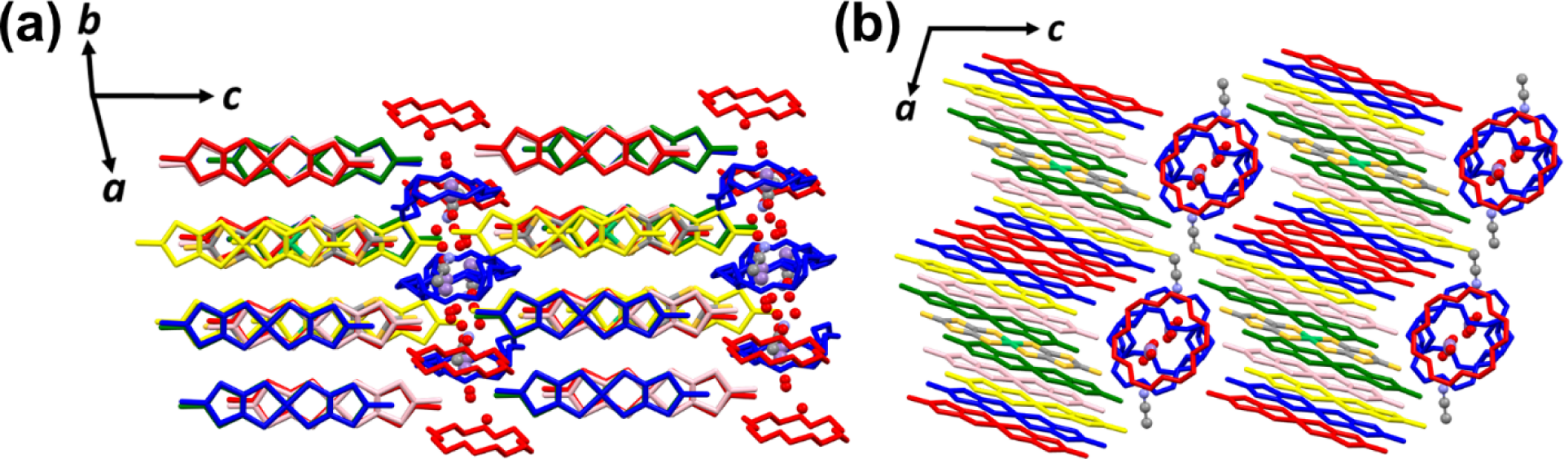
Crystal structure of **1** at 100 K. Manganese ions, acetonitrile, and water are shown as ball-and-stick models, and all other components are shown as sticks. The color of manganese is purple, water is red (oxygen), and acetonitrile is gray and blue-violet (carbon and nitrogen respectively). The two crown ethers (**CE1** and **CE2**) are shown in blue and red, respectively. The 5.5 crystallographically independent [Ni­(dmit)_2_] anions (**A**, **B**, **C**, **D**, **E**, and **F**) are colored blue, green, pink, red, yellow, and element (Ni: light green, C: gray, S: yellow), respectively. Hydrogen atoms are omitted for clarity. (a) Crystal structure viewed along the [Ni­(dmit)_2_] stacking direction. The supramolecular layers and the [Ni­(dmit)_2_] layers are alternately stacked along the *c* axis. (b) Crystal projection along the *b* axis. The supramolecular cations stack along the *b* axis to form channel structures, and the one-dimensional [Ni­(dmit)_2_] columns stack in a direction that makes an angle of approximately 80° with the channels.

The detailed procedures for crystal-structure optimization, including treatment of occupancies and disorder, are provided in the Experimental section of the Supporting Information. Within the ion channels, the two crystallographically independent crown ethers **CE1** and **CE2** stack one-dimensionally along the *b* axis in a repeating sequence of (**CE2**···**CE1**···**CE1**)_*n*_ (Figure 2a). **CE1**, which encapsulates an Mn ion, is disordered at the **C44**–**O5**–**C45** and **O9** sites, whereas the other oxygen and carbon atoms are modeled as common to two conformers. The site occupancies of **O9A** and **O9B** are refined to 0.75 and 0.25, respectively, and the corresponding **CE1** conformers are denoted **CE1A** and **CE1B**, respectively. As **O9** coordinates with an Mn ion, the **O9** disorder correlates with the Mn-site disorder, as described below. In contrast, **C44A**–**O5A**–**C45A** and **C44B**–**O5B**–**C45B** have equal occupancies of 0.50, which indicates that the disorders at **C44**–**O5**–**C45** and **O9** are not correlated. The **C44**–**O5**–**C45** moiety is disordered over two sites in both **CE1A** and **CE1B**, irrespective of Mn coordination, as evidenced by pronounced anisotropic displacement ellipsoids in the crystallographic analysis. The structural features of **CE1** revealed by variable-temperature single-crystal X-ray diffraction are summarized in Figure S2. The **C44**–**O5**–**C45** moiety observed at 100 K undergoes continuous thermal fluctuations; at temperatures ≥ 150 K it was modeled as a single atomic position with a large displacement parameter.

**Figure 2:**
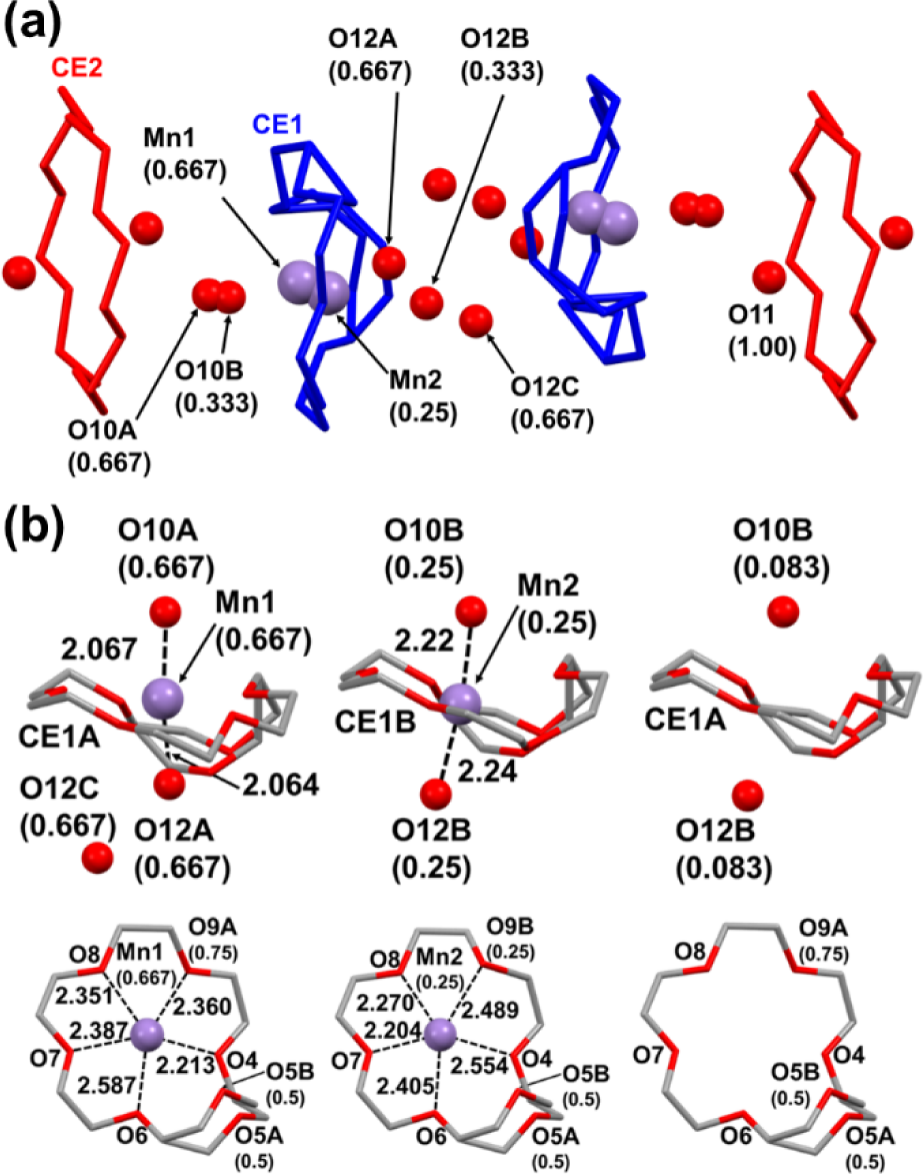
Supramolecular structure of **1**. Carbon, oxygen, and manganese are shown in gray, red, and purple, respectively. The numbers in parentheses indicate site occupancies for each atom, and the numbers adjacent to the dashed lines indicate interatomic distances (Å). (a) View of the supramolecular channel structure along the *c* axis. The crown ethers **CE1** and **CE2** are colored blue and red, respectively. (b) Side and top views of **CE1** with respect to the crown-ether plane. Left, **CE1A** encapsulating **Mn1**. Center, **CE1B** encapsulating **Mn2**. Right, **CE1A** without a manganese ion.

The Mn cations are distributed over two partially occupied sites, **Mn1** and **Mn2**, with refined occupancies of 0.667 and 0.25, respectively. Given the 0.75:0.25 population ratio of **CE1A** and **CE1B**, **Mn2** is plausibly associated with the **CE1B** inclusion site. The 0.75 fraction of **CE1A** comprises a 0.667 fraction that encapsulates **Mn1** and a residual 0.083 fraction of **CE1A** that remains guest-free (Figure 2b). Excluding **O5**, which lies far from the Mn ions, the **Mn1**···O distances to the remaining five oxygen atoms of **CE1A** are 2.213(3), 2.587(2), 2.387(2), 2.351(2), and 2.360(2) Å, respectively. For **Mn2** with **CE1B**, the corresponding **Mn2**···O distances are 2.554(3), 2.405(3), 2.204(3), 2.271(3), and 2.489(8) Å, respectively. Accordingly, each Mn ion is weakly coordinated by five oxygen donors in an approximately equatorial arrangement.

Each Mn ion is coordinated by two water molecules located above and below, designated **O10** and **O12**, respectively. The **O10** site is split over **O10A** and **O10B** with occupancies of 0.667 and 0.333, respectively. It is therefore reasonable to assign **O10A** to **Mn1** and **O10B** to **Mn2**. The **Mn1**···**O10A** distance is 2.067(6) Å and the **Mn2**···**O10B** distance is 2.22(1) Å, which are consistent with the coordination of water to manganese. The **O12** site is disordered over **O12A**, **O12B**, and **O12C** with occupancies of 0.667, 0.333, and 0.667, respectively. The **O12A**···**O12B** and **O12B**···**O12C** separations are 1.273(9) and 1.26(1) Å, respectively, which are too short for simultaneous occupation; therefore, **O12B** cannot coexist with **O12A** or **O12C**. The occupancies indicate that **O12A** and **O12C** are both present in the **Mn1** configuration. The **Mn1**···**O12A** distance is 2.064(6) Å, which is compatible with coordination to **Mn1**. The **Mn1**···**O12C** separation is 4.095(5) Å, which indicates that **O12C** is relatively isolated. In contrast, the **Mn2**···**O12B** distance is 2.24(1) Å, which indicates coordination to **Mn2**. Thus, each manganese center adopts a seven-coordinate environment comprising two axial aqua ligands and five equatorial crown-ether O donors. The **CE1A** conformer without manganese coexists with **O10B** and **O12B**. All Mn···O distances and O–Mn–O angles are listed in Table S1. **O11** of the water molecule interacts with two oxygen atoms of **CE2**, with O···O separations of 2.799(3) and 3.112(3) Å, respectively. Accordingly, **O11** is not located at the ring center and lies 1.596 Å from the mean plane defined by the crown-ether oxygen atoms. Together with the inversion-related water molecule, **O11** sandwiches **CE2** from above and below.

The ionic dynamics of Mn ions within the channels can be inferred from the infrared band near 1105 cm^–1^, which is assigned to the asymmetric C–O–C stretching vibration of [18]­crown-6 (Figure S3 and Table S3). In [18]­crown-6 ion-channel crystals with mobile channel cations, exemplified by [(Li^+^)_0.42_([18]­crown-6)]­[Ni­(dmit)_2_]_2_,^19^ this infrared band is broadened. In contrast, the band for **1** is comparatively sharp, which is consistent with the suppression of ionic motion by the coordination environment formed by [18]­crown-6 and water molecules within the channels.

Five crystallographically independent [Ni­(dmit)_2_] anions (**A**–**E**) and one-half of the [Ni­(dmit)_2_] anion **F** (located on an inversion center) assemble into a one-dimensional columnar structure with a nonuniform stacking sequence. The repeat unit is **A**–**B**–**C**–**D**–**E**–**F**–**E**′–**D**′–**C**′–**B**′–**A**′, where the prime denotes molecules generated by symmetry operations (Figure 3). To the best of the authors’ knowledge, the number of crystallographically independent [Ni­(dmit)_2_] anions observed in **1** is the largest reported among [Ni­(dmit)_2_]-based crystals. The shortest S···Mn separations between sulfur atoms of [Ni­(dmit)_2_] and **Mn1** or **Mn2** are 5.485(1) and 5.039(2) Å, respectively, indicating that no significant magnetic exchange is expected via direct contact between the manganese ions and the [Ni­(dmit)_2_] layer.

**Figure 3:**
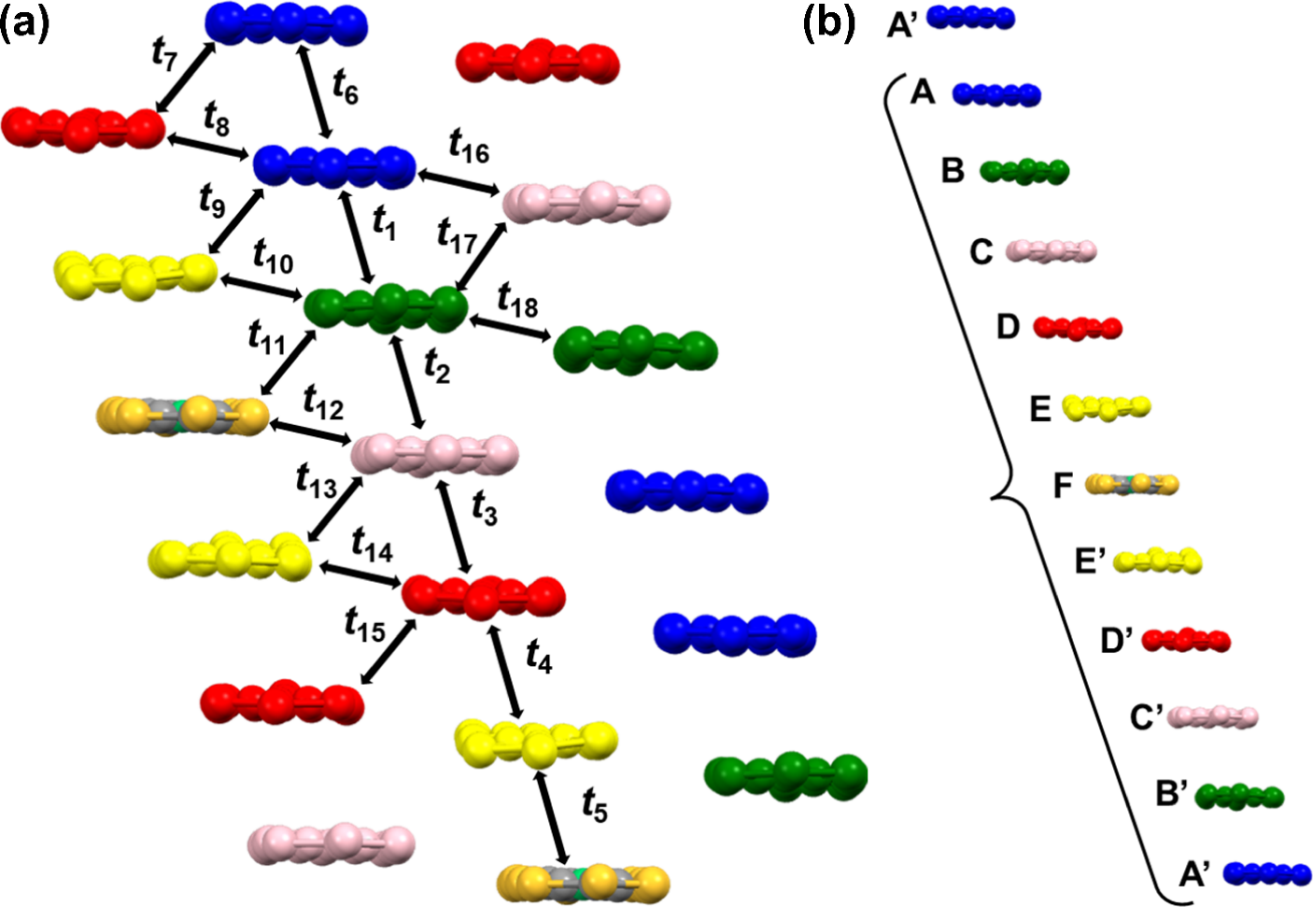
(a) Stacking arrangement of [Ni­(dmit)_2_] molecules. Crystallographically independent molecules are colored as in Figure 1. Intermolecular transfer integrals *t*_1_–*t*_18_ are indicated by arrows, and the corresponding values are listed in Table 1. (b) Repeating unit along a one-dimensional [Ni­(dmit)_2_] stack.

Table 1 lists the transfer integrals between [Ni­(dmit)_2_] anions. Within the column, the transfer integrals between **A**–**B** and **C**–**D** are particularly large: *t*_1_ = 204.2 meV and *t*_3_= 217.4 meV, respectively, whereas the remaining intrastack interactions lie in the range of 10–40 meV, indicating that **A**–**B** and **C**–**D** form strong dimers. In contrast to the ring-overbond overlap in **A**–**A**′, **B**–**C**, and **E**–**F**, the nearly face-to-face arrangement of [Ni­(dmit)_2_] molecules in **A**–**B** and **C**–**D** agrees well with the magnitudes of the transfer integrals. Therefore, the column is regarded formally as being segmented into (**A**–**B**), (**C**–**D**), and (**E**–**F**–**E**′), although the intermolecular separations within the **E**–**F**–**E**′ trimer are comparatively long and the corresponding interactions are weaker than those within the **A**–**B** and **C**–**D** dimers. In the lateral direction, the intercolumn transfer integrals are smaller than those along the stacks, yet relatively strong couplings are found between **B** and **F** and between **D** units, in adjacent columns. These features indicate that [Ni­(dmit)_2_] forms a two-dimensional conducting layer mediated by moderately weak interchain interactions.

**Table 1:** Transfer Integrals between [Ni­(dmit)_2_] Molecules within a One-Dimensional Column (Intrastack) and between Columns (Interstack)

Intrastack	Transfer Integral (meV)	Interstack	Transfer Integral (meV)
*t*_1_ (A–B)	204.2	*t*_7_ (A′–D)	–7.00
*t*_2_ (B–C)	12.16	*t*_8_ (A–D)	–3.60
*t*_3_ (C–D)	217.4	*t*_9_ (A–E)	6.58
*t*_4_ (D–E)	39.78	*t*_10_ (B–E)	2.60
*t*_5_ (E–F)	–8.72	*t*_11_ (B–F)	–21.0
*t*_6_ (A–A′)	–17.33	*t*_12_ (C–F)	–6.97
		*t*_13_ (C–E′)	6.24
		*t*_14_ (D–E′)	3.82
		*t*_15_ (D–D′)	–36.3
		*t*_16_ (A–C′)	–0.715
		*t*_17_ (B–C′)	10.8
		*t*_18_ (B–B′)	–6.97

It is well established that in [Ni­(dmit)_2_]*^n^* ^–^ (*n* = 0, 1, 2), increasing n shortens the π­(C=C) bond while lengthening both σ­(C–S) and σ­(Ni–S) bonds. Therefore, the intramolecular bond lengths (C=C, C–S, and Ni–S) of the [Ni­(dmit)_2_] anions were examined to estimate the molecular charge states present in the crystal; the data are listed in Table S2. Molecules **A**, **B**, **C**, and **D** exhibit similar intramolecular metrics, with mean bond lengths of 1.643–1.646 Å for C=C, 1.699–1.703 Å for C–S, and 2.157–2.158 Å for Ni–S. Compared with reported values for the monovalent [Ni­(dmit)_2_]^−^ complex (C=C: 1.360 Å; C–S: 1.715 Å; Ni–S: 2.166 Å), these molecules show longer C=C and shorter C–S and Ni–S bond lengths. As A–B and C–D form strong dimers, each pair is assigned an average total charge of −1, corresponding to an anion dimer, [Ni­(dmit)_2_]_2_^–^. In contrast, the intramolecular bond lengths of molecules **E** and **F** are 1.388(3) and 1.391(4) Å for C=C, 1.693(1) and 1.691(1) Å for C–S, and 2.1522(4) and 2.1489 Å for Ni–S, respectively, that is, longer C=C and shorter C–S/Ni–S compared to those in **A**–**D**. Thus, **E** and **F** are in higher oxidation states than **A**–**D**. On this basis, the weakly associated **E**–**F**–**E**′ unit is assigned as an anion trimer, [Ni­(dmit)_2_]_3_^–^. This assignment indicates that the 5.5 crystallographically independent [Ni­(dmit)_2_] anions together carry a total charge of −2.5. The Mn cations are disordered over two sites, **Mn1** and **Mn2**, with occupancies of 0.667 and 0.25, respectively. If **Mn1** is taken as trivalent and **Mn2** as divalent, the total positive charge is 0.667 × 3 + 0.25 × 2 = 2.5, which balances the −2.5 charge of the [Ni­(dmit)_2_] sublattice. This charge distribution is consistent with the magnetic measurements described below.

Figure 4 shows the temperature dependence of molar magnetic susceptibility (χ_m_) for **1**, in which χ_m_ *T* decreases with decreasing temperature, indicating antiferromagnetic interactions among the spins. χ_m_ was analyzed by treating the [Ni­(dmit)_2_] sublattice as a one-dimensional Heisenberg antiferromagnetic chain^20,21^ and the crown-ether-encapsulated Mn ions within a Curie–Weiss^22,23^ approximation, using the following expression:

1 $$\chi_{m}=\left(\frac{C_{Mn}}{T-\theta }\right)+\left(\frac{4C_{dmit}}{T}\frac{0.25+0.074975x+0.075235x^{2}}{1+0.9931x+0.172135x^{2}+0.757825x^{3}}\right)$$

where *C*_Mn_ and *C*_dmit_ are Curie constants for Mn ions and the [Ni­(dmit)_2_] sublattice, respectively, and θ is the Weiss temperature. Parameter *x* is defined as *J*/k_B_ *T* where *J* is the magnetic exchange interaction and k_B_ is Boltzmann’s constant. The best agreement was obtained with *C*_Mn_ = 3.13(4) emu K mol^–1^, *C*_dmit_ = 0.97(4) emu K mol^–1^, θ = −0.11(3) K, and *J*/k_B_ = −17.3(10) K. Assuming a Landé *g*-factor of 2, the expected Curie constants are 1.09 emu K mol^–1^ for 0.25 Mn­(II) with *S* = 5/2 and 2.00 emu K mol^–1^ for 0.667 Mn­(III) with *S* = 2, where the sum of 3.09 emu K mol^–1^ agrees well with the fitted *C*_Mn_. The very small θ indicates that the Mn ions are effectively isolated. For the [Ni­(dmit)_2_] chains, one unpaired electron resides on each of the [Ni­(dmit)_2_]_2_^–^ dimers **A**–**B** and **C**–**D**, and one resides on the [Ni­(dmit)_2_]_3_^–^ trimer **E**–**F**–**E**′. The calculated Curie constant of 0.94 emu K mol^–1^ for 5.5 crystallographically asymmetric units also matches the fitting. It is well-known that the magnetic exchange interaction scales with the square of the transfer integral, and the obtained *J*/k_B_ is consistent with the comparatively small interdimer and intertrimer transfer integrals (≈10–40 meV).

**Figure 4:**
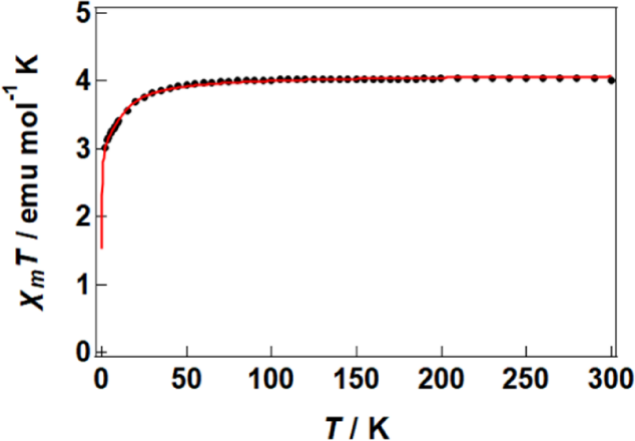
Temperature dependence of χ_m_ *T* derived from the susceptibility measured under a DC field of 5000 Oe. The red solid curve is the fit using Eq 1; values below 2 K are extrapolated predictions down to 0.1 K.

The crystal exhibits semiconducting behavior, consistent with the [Ni­(dmit)_2_] columns constituting a localized-spin system described by a one-dimensional Heisenberg antiferromagnetic chain. Nevertheless, the conductivity at 300 K, measured by the four-probe method along the [Ni­(dmit)_2_] stacking axis, is 4.8 S cm^–1^, a value comparable to that of strongly correlated crystals displaying metallic conduction. The temperature dependence of the conductivity in the range 77–300 K is well described by one-dimensional VRH^24–26^ as follows:

2 $$\sigma \left(T\right)=\sigma_{0}\exp\left[-{\left(\frac{T_{0}}{T}\right)}^{1/2}\right]$$

with σ_0_ = 32.6 S cm^–1^ and *E*_1_ = 0.92 eV (Figure 5). The corresponding effective energy gap at 300 K is *E*_*g*_ = k_B_(*T*_0_/*T*)^1/2^ = 0.055 eV.

**Figure 5:**
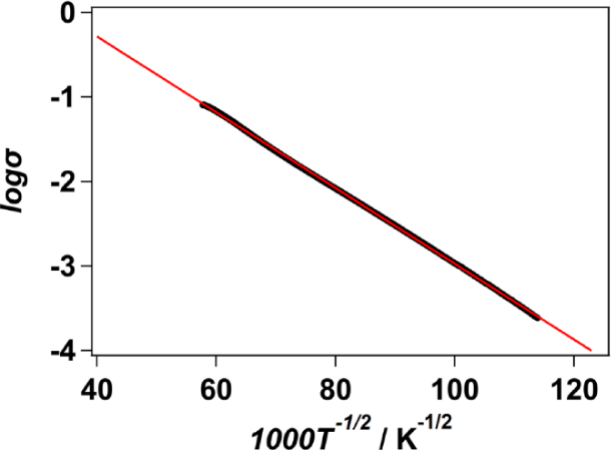
Log σ vs *T* ^–1/2^ plot for crystal **1** measured along the [Ni­(dmit)_2_] stacking axis during cooling from room temperature to 77 K. The red solid line represents a linear fit to eq 2 expressed in logσ–*T* ^–1/2^ coordinates.

VRH conduction is typically associated with disordered systems, but it has also been reported in single crystals such as (Et_4_N)­[Ni­(dmit)_2_] and (Et_4_N)­[Pd­(dmit)_2_]_2_.^27–29^ The latter shows high room-temperature conductivity and is comparable to **1** in that strongly coupled [Pd­(dmit)_2_]_2_ dimers (*t*_intradimer_ ≈ 630 meV) are only weakly coupled within the plane (*t*_in‑plane_ ≈ 25 meV), yielding an effectively two-dimensional network. Despite the crystallographic order, the logarithm of conductivity follows a *T* ^– 1/2^ dependence characteristic of VRH between localized states. A plausible origin is the large thermal motion of the conducting anions, which induces fluctuations in the transfer integrals between [Pd­(dmit)_2_] that define the electronic bands and leads to carrier localization.

The conductivity of a disordered metal is governed by the carrier mobility, which is proportional to the hopping probability Γ between localized states,^30,31^ described as follows:

3 $$\Gamma =\upsilon_{0}\exp\left(-\alpha R-\Delta kT\right)$$

where υ_0_ is a constant of the order of a phonon frequency, α is the decay constant of the localized wave function, and Δ is the energy difference between two localized states separated by a distance *R*. In the standard VRH framework, the hopping distance is selected to maximize Γ. Increasing *R* reduces the typical energy mismatch and is thus thermally favorable, whereas the wave function overlap decreases and the probability diminishes. The optimal hopping radius is determined by the balance of these effects.^32^ In (Et_4_N)­[Pd­(dmit)_2_]_2_, dynamic disorder of [Pd­(dmit)_2_] occurs within an otherwise crystallographically ordered lattice. The thermal motion of the anions induces fluctuations in the transfer integrals, which prevent the formation of a well-defined periodic band structure and yield localized wave functions extending over only a finite number of molecules.^33,34^ The electronic response time is of the order of 10^–15^ s, which is significantly shorter than the phonon period of approximately 10^–12^ s. Therefore, the disorder can be considered effectively static. The resulting localization length is smaller than the usual VRH radius, and the hopping probability is controlled by the temperature dependence of this localization length. As the amplitude of anion vibrations varies with temperature, the localization length changes accordingly. This length sets both the typical overlap in the first exponential factor in eq 3 and the characteristic energy difference in the second factor. In the case of (Et_4_N)­[Pd­(dmit)_2_]_2_, with an anion vibrational amplitude proportional to *T* ^1/2^, the logarithm of the conductivity varies linearly with *T* ^1/2^.

To evaluate the anion vibrations in **1**, for each crystallographically independent [Ni­(dmit)_2_] molecule, the isotropic displacement parameter *U*_eq_ of the terminal thiocarbonyl sulfur atoms was determined over 100–300 K (Figure 6). In all cases, *U*_eq_ increases approximately in proportion to temperature. As the effective root-mean-square amplitude (*u*_rms_) of three-dimensional atomic motion is given by *u*_rms_ = (3*U*_eq_)^1/2^, the temperature dependence of the conductivity of **1** can be understood in terms of VRH through a mechanism analogous to that proposed for (Et_4_N)­[Pd­(dmit)_2_]_2_.

**Figure 6:**
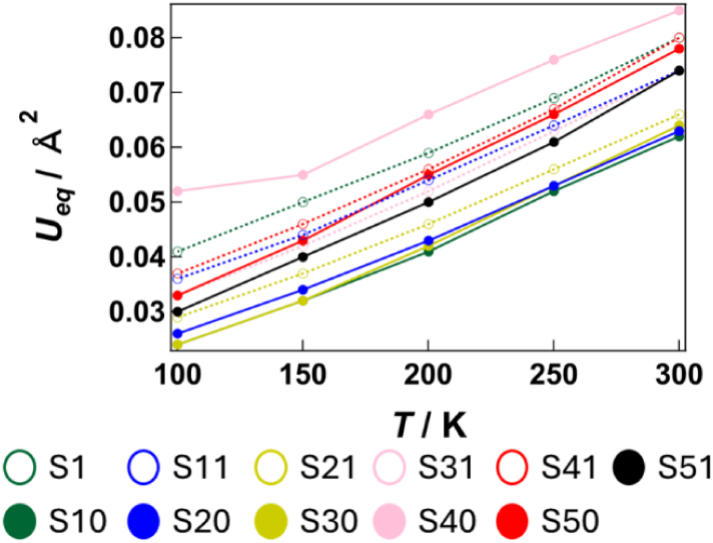
Temperature dependence of the equivalent isotropic displacement parameter (*U*_eq_) for the terminal sulfur atoms of [Ni­(dmit)_2_] molecules. Plot colors follow the color scheme defined for the crystallographically independent [Ni­(dmit)_2_] units in Figure 1. For each molecule, the terminal sulfur atoms **S10**, **S20**, **S30**, **S40**, and **S50** lie on the supramolecular side, whereas **S1**, **S11**, **S21**, **S31**, and **S41** lie on the opposite side.

The atomic displacements within the [Ni­(dmit)_2_] sublattice are strongly influenced by thermal fluctuations of the supramolecular columns. Among the six types of crystallographically independent [Ni­(dmit)_2_] molecules that constitute the one-dimensional column, the *U*_eq_ of the **S40** thiocarbonyl atom in molecule **C** is particularly large over the entire temperature range. Within the crystal, the [Ni­(dmit)_2_] columns are mutually inclined by approximately 80° to the supramolecular columns. Molecule **C** is located where the two columnar arrays are the closest (Figure S4). The **S40** of molecule **C** lies near the disordered **C44**–**O5**–**C45** fragment of **CE1**. In particular, the distance between **S40** and **C44A** in **CE1A** at 100 K is 3.367 Å, which is shorter than the sum of the van der Waals radii of S and C atoms (3.5 Å) (Figure S5). Accordingly, molecule **C** is strongly influenced by the thermal motion of **CE1**, resulting in an enhanced *u*_rms_. In M_*X*_([18]­crown-6)­[Ni­(dmit)_2_]_2_, fluctuations of the alkali-metal ions inside the ion channels strongly affect transport in the [Ni­(dmit)_2_] conducting layers,^19^ whereas in **1**, the fluctuations of the supramolecular channel itself govern the conduction mechanism in the [Ni­(dmit)_2_] layers by modulating the transfer integrals.

## Conclusions

This study constructed Mn_1.83_([18]­crown-6)_3_[Ni­(dmit)_2_]_11_(H_2_O)_7.33_(CH_3_CN)_2_ (**1**), in which one-dimensional [18]­crown-6 channels encapsulating the localized spins of Mn ions coexist with conducting layers formed by [Ni­(dmit)_2_]. Within the channels, Mn^2+^ (0.25 per site) and Mn^3+^ (0.667 per site) are statistically distributed. The crystallographically independent [Ni­(dmit)_2_] molecules organize into strongly interacting dimers **A**–**B** and **C**–**D** and a weakly interacting trimer **E**–**F**–**E**′, and these units assemble into one-dimensional stacks. No direct contact between the Mn spin system and [Ni­(dmit)_2_] was observed. The Mn sublattice follows a Curie–Weiss law with a very low Weiss temperature of −0.11(3) K, whereas the [Ni­(dmit)_2_] sublattice is described by a one-dimensional Heisenberg antiferromagnetic chain with *J*/k_B_ = −17.3(10) K. Furthermore, the conductivity at room temperature is as high as 4.7 S cm^–1^, and its temperature dependence is described by one-dimensional VRH. The thermal motion of the supramolecular columns induces fluctuations in the [Ni­(dmit)_2_] columns; therefore, the electronic wave functions remain localized over a finite number of molecules and VRH conduction emerges. Thus, the thermal fluctuations of the supramolecular columns govern the conduction mechanism of the [Ni­(dmit)_2_] conducting layers.

This supramolecular strategy provides a methodological framework for introducing magnetic ions into [Ni­(dmit)_2_]-based conductors to investigate the cooperative interactions between electrical conduction and magnetism. Beyond the coexistence of spins and carriers, substituting oxygen atoms in [18]­crown-6 with sulfur or nitrogen in thia- or aza-crown analogues could reinforce coupling at the channel–conduction-layer interface via S···S contacts or hydrogen-bond networks. Such control would enable systematic tests, within a common architecture, to evaluate interaction regimes ranging from RKKY-type coupling to stronger interactions that can yield colossal magnetoresistance. Furthermore, the valence distribution and ion/molecular motion within the channels can indirectly tune the charge allocation and stacking in the conducting layers. Thus, systems that permit motion in the channels may enable functionalities such as spin alignment and the potential modulation of the conduction electrons, opening routes to hybrid phenomena based on spin–carrier coupling.

## Experimental Section

### Sample Preparation

Crystals of **1** were obtained by electrocrystallization using a constant-current power supply with a mixed solvent of CH_3_CN/H_2_O (9:1). A solution containing Mn­(ClO_4_)_2_·H_2_O (80 mg, 3.2 × 10^–4^ mol) and [18]­crown-6 (200 mg, 7.57 × 10^–4^ mol), and another solution containing TBA­[Ni­(dmit)_2_] (12.5 mg, 1.80 × 10^–5^ mol), were separately introduced into an H-shaped electrochemical cell. A constant current of 1 μA was applied to platinum electrodes (1 mm diameter) immersed in the cell for 4 d at room temperature (25 °C) to obtain Mn_1.83_([18]­crown-6)_3_[Ni­(dmit)_2_]_11_(H_2_O)_7.33_(CH_3_CN)_2_ (**1**) as black plate-like crystals. Elemental analysis calculations (%) for C_53_H_46.34_NO_12.67_Ni_5.5_S_55_Mn_0.92_: C 20.96, H 1.54, N 0.46; found: C 20.80, H 1.61, N 0.35.

### Structural Analysis

The crystallographic data for **1** were collected using an XtraLAB Synergy diffractometer (Rigaku Corp.) equipped with a single microfocus Mo Kα X-ray radiation source (λ = 0.71073 Å) at 100, 150, 200, 250, and 300 K. Data collection, cell refinement, and data reduction were performed using CrysAlisPRO (Rigaku Oxford Diffraction, 2021). The initial structure was solved using SHELXT software,^35^ and the structural refinement was performed using the full-matrix least-squares method on *F* ^2^ with the Olex2 package.^36^ All non-hydrogen atoms were refined anisotropically. The selected crystallographic data are listed in Table S4.

In the crystal structure analysis, six distinct [Ni­(dmit)_2_] components were assigned: five full molecules (labeled **A**–**E**) and one-half-molecule (labeled **F**), revealing the presence of 5.5 [Ni­(dmit)_2_] anions within the crystallographic asymmetric unit. Additionally, one complete unit and one-half-unit of the [18]­crown-6 molecule were identified, labeled **CE1** and **CE2**, respectively. Manganese ions, the **CE1** and **CE2** crown ether units, and solvent molecules formed a supramolecular channel structure. Residual electron density showed maximum density within the **CE1** ring, with smaller residual densities observed in the **CE2** ring and within the one-dimensional channel. A single Mn ion was initially assigned within the **CE1** ring. However, initial refinement at a single Mn position yielded a large atomic displacement parameters (ADPs) and residual electron density around the Mn site that was larger than that around **CE2**. This suggested disorder in the Mn ion. This disorder was resolved by modeling **Mn1** and **Mn2** with occupancies of 0.6667 and 0.25, respectively, yielding the optimal structure. These values satisfied the paramagnetic spins of the salt. **CE1** exhibited significantly larger ADPs compared to those of **CE2** and the [Ni­(dmit)_2_] anion, suggesting static/dynamic disorder. In the 100 K structure, disordered O atoms of **CE1** were observed at distances of approximately 2.4–2.5 Å from **Mn1** and **Mn2**, which is within the coordination range. Values obtained by refining the site occupancy using the FVAR command almost matched the Mn site occupancy and showed little temperature dependence, leading to their interpretation as **O9A** and **O9B** atoms coordinated to disordered Mn ion. Disorder was also observed in the **C44A**–**O5A**–**C45A** and **C44B**–**O5B**–**C45B** fragments of **CE1**. The site occupancies for these fragments were nearly equivalent, suggesting they belong to the **CE1** ring not coordinated to an Mn ion. Among the Mn-coordinated H_2_O molecules, **O10A** and **O10B** were initially assigned as a single oxygen atom (**O10**) in the initial structure. This resulted in a large electron density remaining near the **O10** atom. One of the distances between **O10** and the two disordered Mn atoms (**O10**···**Mn1** and **O10**···**Mn2** distances) was short, at approximately 1.6 Å. Therefore, **O10** was interpreted as the disorder of **O10A** and **O10B**, and the occupancy ratios of **O10A** and **O10B** were optimized to 0.6667 and 0.3333, respectively. The residual electron density in the vicinity of the **CE2** site and within the one-dimensional channel was interpreted and assigned to H_2_O and CH_3_CN solvent molecules. Because X-ray diffraction cannot reliably locate hydrogen atoms, it is generally difficult to distinguish a neutral water molecule from an oxonium ion (H_3_O^+^). In the present structure, the charge estimated from the [Ni­(dmit)_2_] bond metrics together with the Mn oxidation states inferred from site occupancies (Mn^2+^/Mn^3+^) satisfies charge neutrality when O11 is treated as neutral H_2_O. Assigning O11 as H_3_O^+^ would introduce excess positive charge not supported by these independent valence indicators; therefore, O11 is modeled as a water molecule.

### Calculation of Transfer Integrals

The transfer integrals (*t*) between [Ni­(dmit)_2_]^−^ anions were calculated within the tight-binding approximation using the extended Hückel molecular orbital method, in which the lowest unoccupied molecular orbital (LUMO) of the [Ni­(dmit)_2_]^−^ molecule was employed as the basis function. Semiempirical parameters for the Slater-type atomic orbitals were obtained from the literature.^37^ The *t* values between pairs of molecules were estimated to be proportional to the overlap integral (*s*): *t* = −10 *s* (eV).

### IR Spectroscopy

A mixture of KBr and the sample crystal was ground until homogeneous and then pressed into pellets. The IR spectra of the pellets were recorded using a FT/IR-4700 spectrometer (JASCO Inc.) in the range of 400–7800 cm^–1^.

### Electrical Conductivity

The temperature dependence of the electrical resistivity of single crystals of **1** was measured using the DC two-probe method along the [Ni­(dmit)_2_] stacking direction. Gold electrodes were formed with gold paste, to which 10-μm-diameter gold wires were attached. Measurements were performed under vacuum in a cryostat (Iwatani Corporation). A Keysight electrometer (B2985A) was used to apply a DC bias of 0.2 V and to record the current.

### Magnetic Properties

Magnetic susceptibility measurement data were measured using an MPMS-5S superconducting quantum interference device (SQUID) magnetometer (Quantum Design Inc.) from 2–300 K. The polycrystal samples were placed in a polyethylene wrap under a DC magnetic field of 5 kOe. The temperature-independent diamagnetic component, χ_0_ = –1.74 × 10^–3^ emu mol^–1^, was subtracted during the fitting step during data analysis.

## Supplementary Material



## Notes

Experimental sections; crystal structures; IR spectra; crystallographic data of **1** (PDF)
