# Supplementary material for: Supramolecular Ion-Channel Engineering of Spin–Charge Coexistence in a [Ni(dmit)2] Conductor Hosting Mixed-Valence Mn Cations
Source: Inorg Chem. 2026 Feb 5;65(6):3745–53. doi: 10.1021/acs.inorgchem.6c00118 (PMC12914632; doi:10.1021/acs.inorgchem.6c00118)
Supplement: Supplementary file 1 [file ic6c00118_si_001.pdf]

Supporting Information

Supramolecular Ion-Channel Engineering of Spin–Charge Coexistence in a $[Ni(dmit)_2]$ Conductor Hosting Mixed-Valence Mn Cations

Daisuke Ishikawa[a], Jun Manabe[a], Masato Haneda[a], Kiyonori Takahashi[b], Takayoshi Nakamura[a,c]*, Sadafumi Nishihara[a,d,e]*

*E-mail: tnakam@hiroshima-u.ac.jp (Takayoshi Nakamura)

*E-mail: snishi@hiroshima-u.ac.jp (Sadafumi Nishihara)

[a]Department of Chemistry, Graduate School of Advanced Science and Engineering, Hiroshima University, 1-3-1, Kagamiyama, Higashi-hiroshima, Hiroshima, 739-8526, Japan.

[b]Department of Chemistry, Kumamoto University,2-39-1 Kurokami, Chuo-ku, Kumamoto 860-8555, Japan.

[c]Research Institute for Electronic Science, Hokkaido University, N20W10, Kita-ku, Sapporo 001-0020, Japan.

[d]Chirality Research Center (CResCent), Hiroshima University, 1-3-1, Kagamiyama, Higashi-hiroshima, Hiroshima, 739-8526, Japan.

[e]Precursory Research for Embryonic Science and Technology (PRESTO), Japan Science and Technology Agency, 4-1-8, Honcho, Kawaguchi, Saitama, 332-0012, Japan.

Table of contents

1. **Crystal structure of 1** **3**

2. **IR spectrometry** **5**

3. **Disorder of 1** **6**

4. **Crystallographic data** **7**

5. **References** **8**

1. Crystal structure of 1

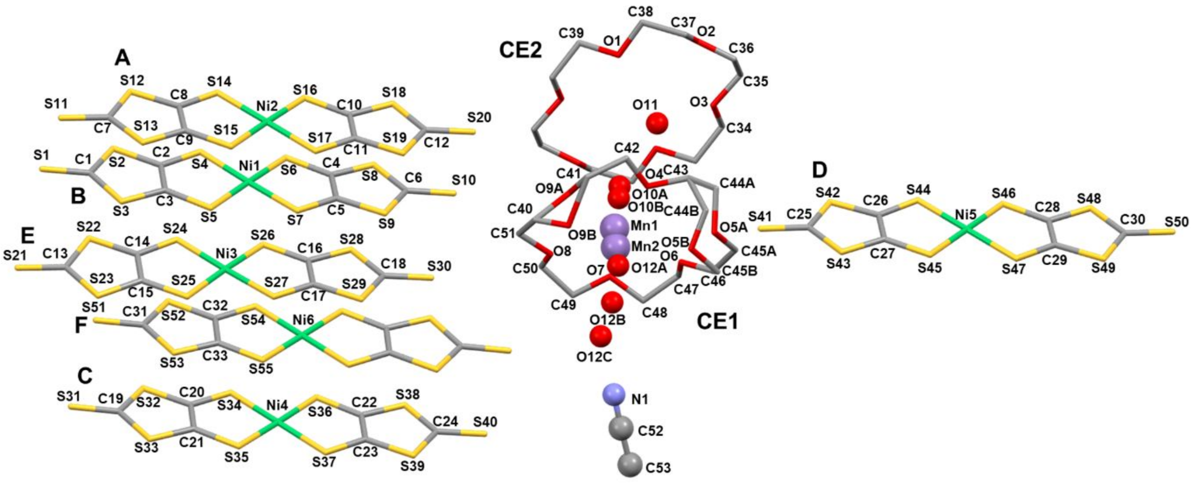

Figure S1. Crystallographically independent constituent. Ni(dmit)$_2$ anions and crown-ether molecules are shown as sticks; all other species are depicted in a ball-and-stick representation. Hydrogen atoms are omitted for clarity.

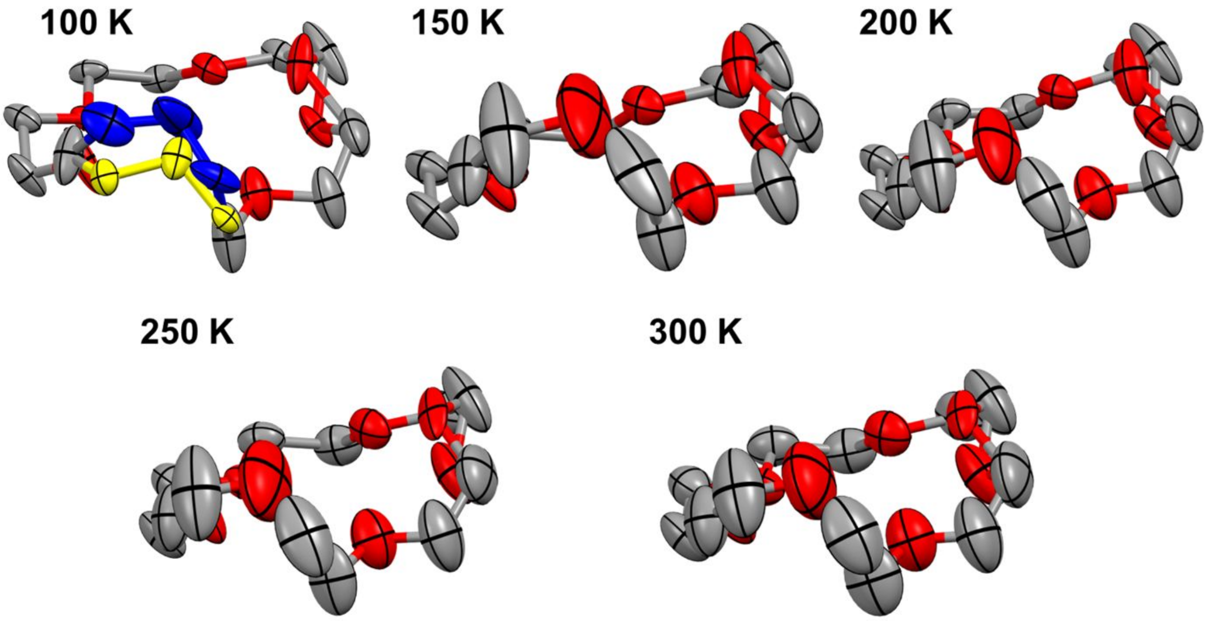

Figure S2. Structures of **CE1** at various temperatures. Carbon atoms are shown in gray and oxygen in red; the disordered components are colored yellow (**CE1A**) and blue (**CE1B**). Models are depicted as anisotropic displacement ellipsoids and sticks. At temperatures ≥150 K, the C44–O5–C45 fragment could not be satisfactorily refined with a disorder model, yet it persistently shows pronounced thermal (librational) motion.

Table S1. Mn–O distances for oxygen atoms from the crown ether and water, and corresponding O–Mn–O angles.

	Distance (Å)		Angle (°)
Mn1-O4	2.213(3)		
Mn1-O6	2.587(2)		
Mn1-O7	2.387(2)		
Mn1-O8	2.351(2)		
Mn1-O9A	2.360(2)		
Mn1-O10A	2.067(6)	O10A-Mn1-O12A	170.2(2)
Mn1-O12A	2.064(6)		
Mn2-O4	2.554(3)		
Mn2-O6	2.405(3)		
Mn2-O7	2.204(3)		
Mn2-O8	2.271(3)		
Mn2-O9B	2.489(8)		
Mn2-O10B	2.22(1)	O12B-Mn2-O12B	171.5(5)
Mn2-O12B	2.24(1)		

Table S2. Average bond length of Ni(dmit)$_2$ and antibonding sites in the LUMO. Within a single molecule, site a appears twice, while sites b and c each appear four times; the average bond length is taken as the mean of these respective bond lengths. Ni–S bond lengths for the Ni(dmit)$_2$ molecule **F** are reported without standard uncertainties because they were not output by the refinement.

	C=C	C-S	S-Ni
A	1.383(3)	1.699(1)	2.1570(4)
B	1.381(3)	1.703(1)	2.1580(4)
C	1.382(3)	1.699(1)	2.1569(4)
D	1.382(3)	1.703(1)	2.1572(4)
E	1.388(3)	1.693(1)	2.1522(4)
F	1.391(4)	1.691(1)	2.1489
$[Ni(dmit)_2]^-$ [1]	1.360	1.715	2.166

2. IR spectrometry

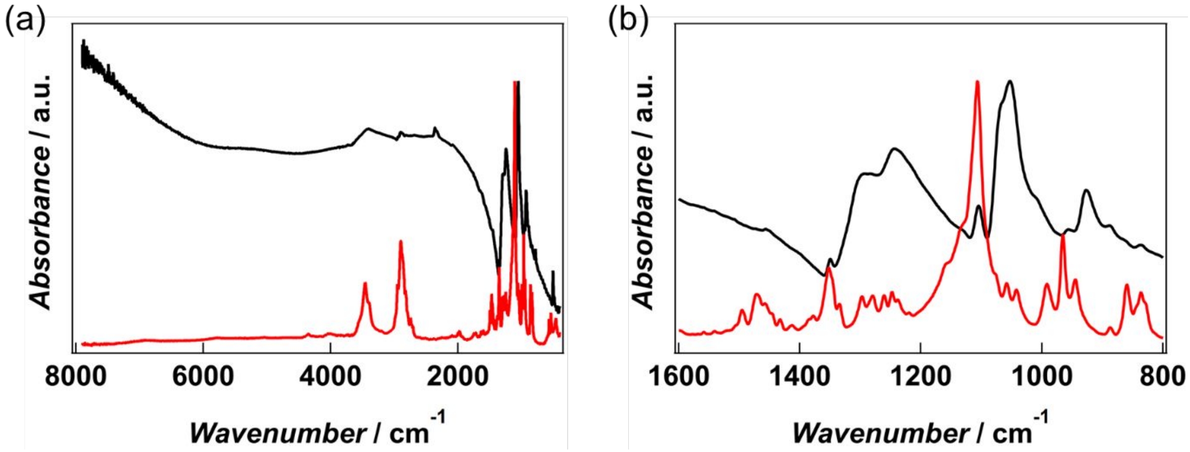

Figure S3. IR spectra of **1** (black) and 18c6 without any inclusion over the ranges of (a) 8000–400 cm^{-1} and (b) 1600–800 cm^{-1}. The half-width at half maximum of the C–O–C asymmetric stretching vibration peak near 1105 cm^{-1} is 12.5 cm^{-1} (compared to 25.1 cm^{-1} for 18c6 without any inclusion), indicating a reduction in the degrees of freedom.

Table S3. Assignment of each IR vibrational mode for **1.**

	Wavenumber (cm^{-1})
C=C stretching	1350
-CH_2 vibration (wagging, twist)	1244, 1290
C-O-C stretching (asymmetric)	1105
C=S stretching	1053
C-O-C stretching (symmetric)	926

3. Disorder of 1

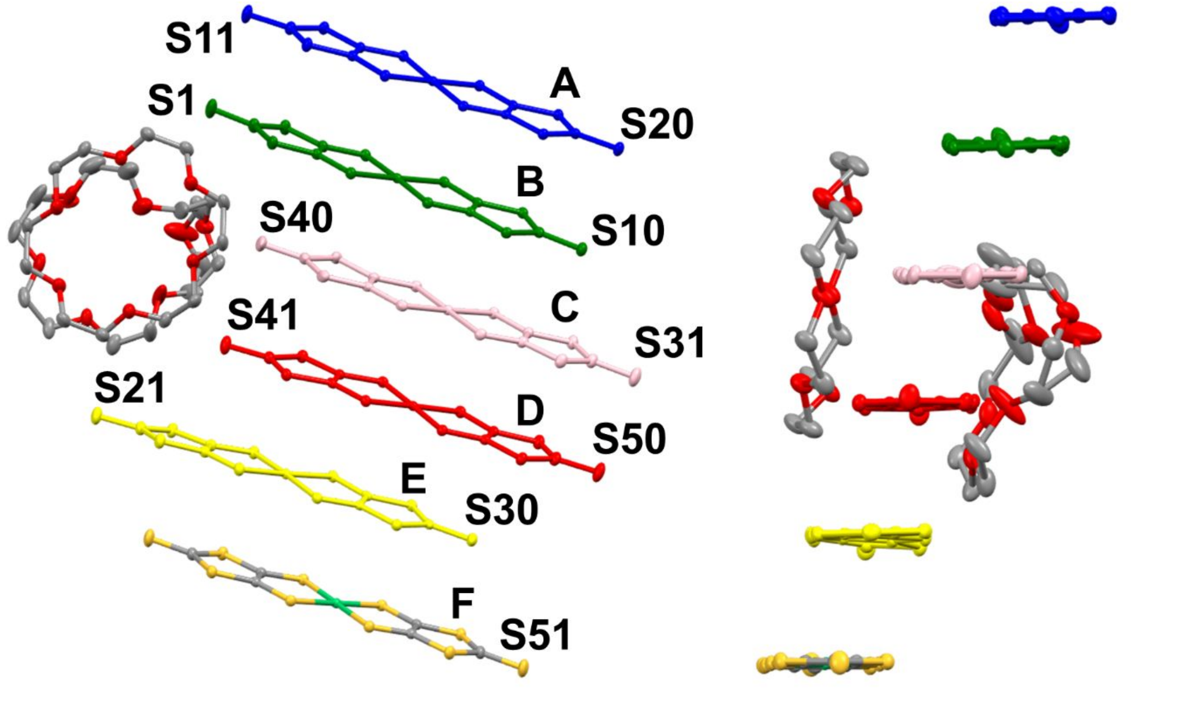

Figure S4. Single [Ni(dmit)$_2$] column and one-dimensional array of 18-crown-6 ethers. The positions of the supramolecular cations relative to the [Ni(dmit)$_2$] column are clearly indicated; the [Ni(dmit)$_2$] molecule closest to the disordered site of **CE1** is molecule **C**. For clarity, Mn ions and solvent molecules are omitted.

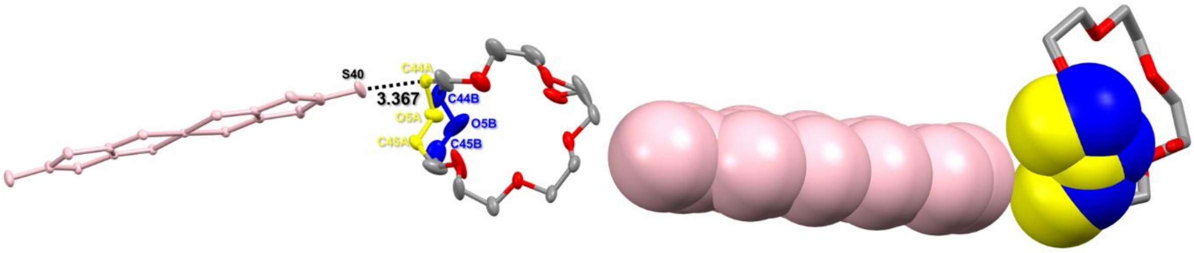

Figure S5. Contact between [Ni(dmit)$_2$] (**C**) and the disordered site of **CE1**. Given the van der Waals radii of carbon (1.70 Å) and sulfur (1.80 Å), a weak contact between **S40** and **C44A** is observed at 100 K.

4. Crystallographic data

Table S4. Crystallographic data for **1**.

Formula	$C_{53}H_{43.667}Mn_{0.917}NNi_{5.5}O_{12.667}S_{55}$
Formula Weight	3041.72
Temperature/K	99.8(5)
Crystal system	triclinic
Space group	P-1
a/Å	15.30640(10)
b/Å	16.8734(2)
c/Å	22.6782(2)
α/deg	91.5810(10)
β/deg	103.8900(10)
γ/deg	116.1730(10)
Volume/Å^3	5042.61(9)
Z	2
ρ_{calc}/g cm^{-3}	2.003
μ/mm^{-1}	2.310
F(000)	3064
Crystal size/mm^3	0.36×0.31×0.05
data range Θ/deg	1.872 to 31.131
limits h, k, l	$-20<h<21$
	$-24<k<24$
	$-31<l<31$
Reflections collected	132927
Independent reflections	26980
Data/restraints/parameters	26980/20/1248
Goodness of fit	1.044
R_1, wR_2 [$I>\sigma(I)$]	0.0348, 0.0849
R_1, wR_2 [all data]	0.0453, 0.0890
Largest diff. peak/hole/e Å^{-3}	1.265/−0.832

5. References

1. Yang, H.; Liu, J.-L.; Zhou, L.-C.; Ren, X.-M. Experimental and theoretical investigation of the magnetic and photoconductive nature of a novel two-dimensional, mixed-valence bis(2-thioxo-1,3-dithiole-4,5-dithiolato)nickelate molecular solid. *Inorg. Chem. Front.* **2014**, *1*(5), 426–433. DOI: 10.1039/C3QI00106G.
